# Quality improvement in long-term care settings: a scoping review of effective strategies used in care homes

**DOI:** 10.1007/s41999-020-00389-w

**Published:** 2020-09-04

**Authors:** Neil H. Chadborn, Reena Devi, Kathryn Hinsliff-Smith, Jay Banerjee, Adam L. Gordon

**Affiliations:** 1 Division of Medical Science and Graduate Entry Medicine, School of Medicine, University of Nottingham, Nottingham, UK; 2 NIHR Applied Research Collaboration East Midlands, Nottingham, UK; 3 School of Healthcare, University of Leeds, Leeds, UK; 4 Faculty of Health and Life Sciences, De Montfort University, Leicester, UK; 5 School of Life Sciences, University of Leicester, Leicester, UK

**Keywords:** Nursing home, Care home, Quality improvement, Scoping review, Older people

## Abstract

**Purpose:**

We conducted a scoping review of quality improvement in care homes. We aimed to identify participating occupational groups and methods for evaluation. Secondly, we aimed to describe resident-level interventions and which outcomes were measured.

**Methods:**

Following extended PRISMA guideline for scoping reviews, we conducted systematic searches of Medline, CINAHL, Psychinfo, and ASSIA (2000–2019). Furthermore, we searched systematic reviews databases including Cochrane Library and JBI, and the grey literature database, Greylit. Four co-authors contributed to selection and data extraction.

**Results:**

Sixty five studies were included, 6 of which had multiple publications (75 articles overall). A range of quality improvement strategies were implemented, including audit feedback and quality improvement collaboratives. Methods consisted of controlled trials, quantitative time series and qualitative interview and observational studies. Process evaluations, involving staff of various occupational groups, described experiences and implementation measures. Many studies measured resident-level outputs and health outcomes. 14 studies reported improvements to a clinical measure; however, four of these articles were of low quality. Larger randomised controlled studies did not show statistically significant benefits to resident health outcomes.

**Conclusion:**

In care homes, quality improvement has been applied with several different strategies, being evaluated by a variety of measures. In terms of measuring benefits to residents, process outputs and health outcomes have been reported. There was no pattern of which quality improvement strategy was used for which clinical problem. Further development of reporting of quality improvement projects and outcomes could facilitate implementation.

**Electronic supplementary material:**

The online version of this article (10.1007/s41999-020-00389-w) contains supplementary material, which is available to authorized users.

## Key summary points

**Aim:**

To review quality improvement in care homes and identify quality improvement approach, process evaluation and resident outcomes.

**Findings:**

Seventy five articles were included which described a variety of quality improvement approaches and various methods of process evaluation addressing various clinical problems. Some studies showed benefits to health outcomes, but it was not possible to synthesise due to diversity of data.

**Message:**

Future quality improvement should apply structured reporting of quality improvement initiatives and resident-level interventions in order that findings can by synthesised and implemented.

**Electronic supplementary material:**

The online version of this article (10.1007/s41999-020-00389-w) contains supplementary material, which is available to authorized users.

## Introduction

433,000 people live in UK care homes for older people [1]. Care homes is the generic term for long-term care facilities including both residential homes and nursing homes. In England there are 4400 nursing homes and 11,400 residential homes. In both settings, the bulk of care is provided by care workers, but nursing homes have at least one resident nurse on site at all times. For residential homes, nursing care is provided through in-reach by the National Health Service (NHS) [2, 3]. All UK care homes, even residential homes, meet the international definition of nursing home [4]. Both types of care homes look after people with advanced frailty, 75% have dementia and all have significant functional dependency. Multimorbidity and polypharmacy are common [5]. The average life expectancy for nursing home residents is 1 year and for those in a residential home is 2 years [6].

There is considerable variation in how care delivery is structured in UK care homes and this leads to variability in the quality of care [7]. Clinical governance is complex and negotiated, with care home providers responsible for routine care provision, whilst the NHS, particularly general practitioners, are accountable for medical care provided. This can lead to confusion and uncertainty about who has responsibility for some aspects of care [8]. Only recently, during the COVID-19 pandemic, has a “clinical lead role” been established for a health-care professional to support care homes—however, this is loosely specified and falls someway short of the rigid lines of accountability seen with medical directors and elderly care Physicians for nursing homes in the USA and Netherlands, respectively [9]. There is increasing recognition of the interdependence of the care home sector and the much smaller acute hospital bed base [10]. These observations, coupled to increased emphasis on integration of health and social care by central government [11], have led to a number of initiatives to improve quality of care in care homes [12–14]. However, the extent and level of development of quality improvement (QI) in care homes has not been well described.

Care homes differ from hospitals in terms of structure, function, client and staff groups. For this reason, principles of quality improvement (QI) which are well established in hospitals will need at least adaptation to work within the care home setting [15]. Meanwhile, there is sufficient similarity between care homes in different countries [4, 16], to mean that principles of QI that work in institutional long-term care homes may be similar between nations.

This review aimed to provide an overview of quality improvement projects in care homes, to establish the current extent of internationally reported QI projects in care homes, describe the strategies used, the occupational groups involved, and the outcomes reported. We defined a QI intervention, based on a definition from the US Agency for Healthcare Research and Quality as “a change process in health care systems, services, or suppliers for the purpose of increasing the likelihood of optimal clinical quality of care, measured by positive health outcomes for individuals and populations” (p1 [17]).

## Method

We carried out a systematic search of academic and grey literature databases, anticipating that quality improvement projects may be reported both within and outside academic literature. For formal academic publications, we searched Medline, CINAHL, Psychinfo and ASSIA. For grey literature, we searched OpenGrey, the Healthcare Management Information Consortium (HMIC) database, the National Institute for Health and Care Excellence (NICE) database and Social Care online.

We used search terms to capture articles about quality improvement, such as “Quality Improvement”, “Quality Indicators, Health Care” or “Health Services Research”. We also included terms to identify specific quality improvement strategies, such as “PDSA”, “Model for Improvement” and “Six Sigma”. Finally, to retrieve articles on care homes we included a search approach established through a recent consensus exercise [18], including terms such as “Nursing Home”, “Long-term Care”, “Care Home”, “Residential Home”, “Residential Facility”, “Institutional Care”, “Skilled Nursing Facility”, “Institutionalisation”, “Care Facility” and “Homes for the Aged”. An example search string of how these were applied in the Medline database is summarised in Appendix 1.

Databases were searched from the year 2000 up until 2019. The start date was chosen because of a previous mapping review which showed very little care home research published prior to this date [19] and because of the recency with which QI has become a focus of interest in care homes. Inclusion criteria were that articles had to describe work undertaken in care homes for older people (65 years and older) and to describe QI as change management, rather than describing a method for gaining new knowledge about the resident-level intervention itself (i.e. a research protocol). Articles describing specific quality improvement strategies, such as quality improvement collaboratives (QICs) [20], or plan-do-study-act (PDSA) cycles [21] were included. Articles describing end-of-life care in care homes were included.

Articles were excluded where they focused on projects for temporary residents of care homes, such as those receiving respite and intermediate care, because these are paid for and organised differently from long-term care. Projects focusing on improvement of hospital admission and discharge pathways, on care homes for children, on those with learning disabilities, or on hospices were excluded. Also excluded were research studies where the focus was on knowledge generation about the clinical intervention itself; where the intervention was tightly specified and protocolised, as these would not shed light on the process of implementing the intervention within local contexts and involving staff teams. Title and abstract screen was conducted by the first reviewer (NC) and articles were divided randomly between three second reviewers (RD, KHS, AG). Selection on the basis of full article and also data extraction were conducted by a second reviewer in conjunction with the first reviewer (NC), where disagreements were resolved by discussion, until consensus was reached. An audit trail was maintained as authors independently and sequentially conducted initial data extraction for all sources. Testing was conducted to ensure agreement and testing of the extraction form and cross-checking of data occurred throughout the process with two members of the team.

To adopt a consistent approach, we described data on QI strategies (structured approaches to change management) separately from the resident-facing interventions which they sought to implement. This enabled us to understand both the range of organisational approaches adopted and the breadth of changes to resident care described. Data extraction forms were developed (see Appendix 2) to collate, firstly, the following information about the quality improvement strategy (name of the QI strategy, number of staff, occupational groups involved, number of participating care homes, any control of comparator, and which process or outcome measures were reported), and, secondly, the resident-level intervention (number of participants, intervention descriptor, any control or comparator, outcome measures and results). Quality appraisal was not a selection criterion because the scoping review aimed to report on the breadth of literature. Instead, methodological weaknesses were captured and discussed. A descriptive synthesis will be performed on the extracted data; firstly, data evaluating the QI strategy (change management) will be synthesised, that is data at staff, team or organisational level. Secondly, data reporting impacts or outcomes at resident level will be synthesised. This report has followed the guidance on reporting scoping reviews: the extended PRISMA guideline as described in Appendix 3 [22].

## Results

**Fig. 1:**
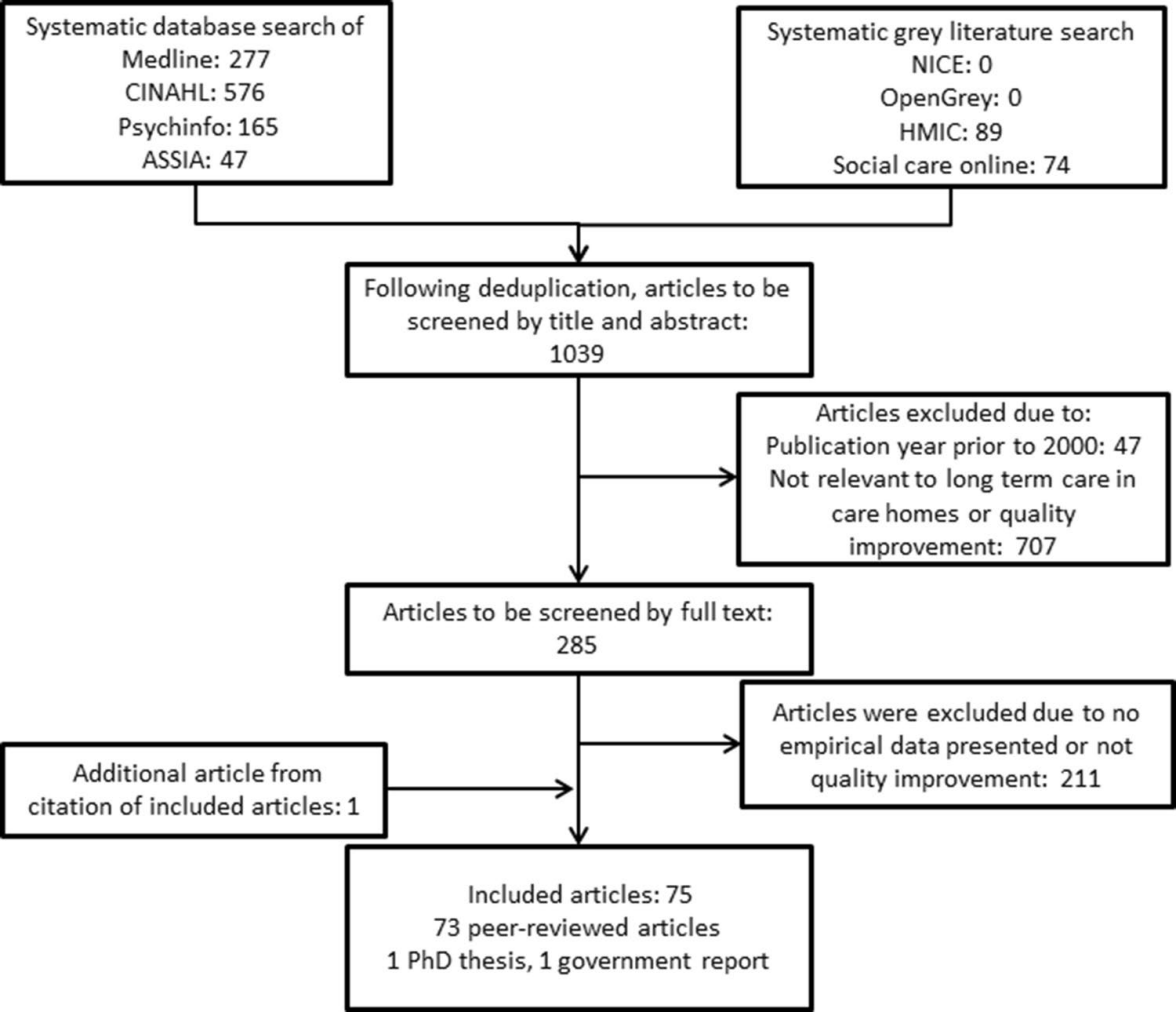
PRISMA flow diagram of articles retrieved from search, screened and selected for review

One thousand and sixty-fifth 1065 were retrieved from academic bibliographic databases and a further 163 from grey literature. A PRISMA diagram summarising de-duplication and screening is shown in Fig. 1. 75 articles were included in the review, with only two articles being grey literature (a list of excluded articles is available on request to the authors). Six studies have multiple articles, so 65 studies are reported [12, 15, 23–95].

The publication rate increased over each complete 5-year period included in the review. For example, 6 articles were published during the 2000–04 period, compared with 27 articles between 2010- and 2014. The majority of articles came from the USA (*n* = 49), with smaller contributions from Canada (*n* = 7), UK (*n* = 7), Australia (*n* = 3), the Netherlands (*n* = 3) and other European countries.

The majority of papers (*n* = 70) described or evaluated a single quality improvement project. Most studies (*n* = 35) reported single arm intervention studies with comparison of quantitative data captured about clinical outcomes before and after the quality improvement project was carried out [23–57]. Qualitative studies were the second largest group (*n* = 19) [12, 23, 27, 30, 58–72], including the following methods: participatory action research (*n* = 2) [12, 63], observational (*n* = 4) [62, 73–75], interviews (*n* = 1) [64], questionnaire (*n* = 1) [69]. Eleven studies were interventional studies with a comparator arm, with quantitative outcome measures, including; 8 randomised controlled trials, all of which were cluster randomised at care home level [76–83]. Four were non-randomised controlled trials [84–87]. Five articles drew comparison between multiple QI initiatives or multiple implementation sites; these papers included reports of characteristics of implementation and descriptive statistics, for example of quality indicators [73–75, 88, 89]. 15 articles came from six studies that published multiple papers about a single QI intervention, for example protocol articles, intervention development and analysis of a subset of the data. These were not duplicate publications, but rather publications of complementary descriptions and analyses of, often complex, QI projects. The six studies were SCOPE (Safer care for older persons (in residential) environments) [23, 65, 90], Connect for quality [77, 91], INTERACT (Interventions to Reduce Acute Care Transfers) [59, 79, 92, 93], PROSPER (PROmoting Safer Provision of care for Elderly Residents) [12, 15, 72].

We found only five articles which applied standardised reporting guidelines. Four followed the CONSORT guidance for trials [75, 77, 79, 81] and one used the SQUIRE 2.0 quality improvement checklist [25]. Due to the diversity of methods reported within studies, it was not possible to use a formal tool to appraise quality across all articles. The review team did, however, identify weaknesses in study design and reporting. We found 35 studies either had deficiencies in methods [24, 30, 33, 37, 39, 50–55, 57, 69, 70, 73–75, 82–84] or were descriptive without process or outcome data [12, 15, 43–50, 61, 62, 66, 67, 71, 72, 76, 88, 89]. Weaknesses included small sample size (for example, one care home sampled), no comparator or baseline, and number of participants not reported. Several studies reported the number of beds and identified the number of cases per bed, making it difficult to elucidate the numbers of participants in the study. Selection bias was identified in three studies, where underperforming care homes were recruited [33, 57, 82]. This represents a tension in QI literature, where legitimate targeting of QI interventions may limit the generalisability of findings to care homes which are already delivering high quality care.

Considering quality improvement strategies adopted, five studies reported using quality improvement collaboratives, or breakthrough series [30, 50, 57, 84, 93], nine studies reported using ‘Plan Do Study Act’ (PDSA) or similar iterative change management [24, 25, 32, 40, 50, 51, 68, 75, 78] and one reported using the Toyota method, also known as kaizen or continuous improvement [35]. Other studies described components quality improvement, but without specifying a particular strategy. Components included education about clinical conditions or care (*n* = 19) [28, 31, 33, 36, 38, 43, 46, 49, 52, 55, 58, 60, 65, 67–69, 83, 86, 94], care pathway development (*n* = 12) [31, 39, 46, 55, 56, 67, 73, 76, 77, 85, 86, 91], audit and feedback (*n* = 14) [28, 33, 37, 49, 58, 61, 76, 77, 81, 87, 91, 93–95], changes to multidisciplinary team working (*n* = 11) [28, 38, 40, 41, 48, 71, 79, 85–87, 95], and enabling peers or champions to lead QI initiatives (*n* = 10) [28, 36, 38, 63, 65, 69, 77–79, 91].

Thirty-eight studies engaged with a QI expert to oversee and deliver the QI approach in the care home setting. Furthermore, 14 of these studies reported that the QI external expert was not engaged with the study team (i.e. QI consultants). In 17 studies, a member of the study team acted as external facilitator. Nine studies required care homes to appoint their own local facilitator or champion. Two studies describe a collaboration between external facilitators in conjunction with care home staff facilitators.

The occupational groups taking part in QI improvement initiatives were predominately nurses (in 46 studies), care assistants (in 28 studies) and care home managers or administrators (in 25 studies) (see Table 1). Other occupational groups were rehabilitation therapists (including physiotherapists and occupational therapists) (in 17 studies), doctors (in 10 studies), social workers (in 10 studies), directors of nursing (or care) (in 9 studies), dietary staff (including dieticians, nutritionists and chefs) (in 5 studies) and pharmacists (in 3 studies). 29 studies described teams of multiple occupational groups or professions (3 or more staff groups) taking part in the QI intervention (see right hand column in Table 1). Five studies described multiprofessional teams, or that all staff of the care home participated in QI, but it is unclear which occupational groups these descriptions may include [33, 50].

**Table 1:** Occupational groups involved in QI initiatives described in studies

Occupational category	Number of studies	References (1 or 2 occupational groups)	References (3 or more occupational groups)
Nurses (registered)	46	[27, 37, 38, 45, 46, 51, 55–57, 62, 71, 75, 79–81, 85, 88]	[23, 25, 29, 30, 35, 36, 40–44, 52, 54, 58, 63, 66–70, 76, 77, 82–87, 93]
Care assistant (non-registered)	28	[12, 24, 37, 38, 51, 55, 60, 71, 79, 81]	[23, 29, 35, 40, 43, 58, 63, 66, 68–70, 77, 82, 83, 85–87, 93]
Administrator/manager	25	[12, 24, 34, 56]	[23, 29, 30, 35, 40, 42, 44, 54, 58, 63, 67–70, 77, 82–84, 86, 87, 93]
Rehabilitation therapists	17		[23, 29, 35, 40–42, 44, 54, 63, 67, 69, 76, 77, 84–87]
Doctor	10	[28, 31, 53, 94]	[25, 52, 58, 76, 77, 93]
Social worker	10		[25, 29, 30, 36, 40, 52, 54, 69, 86, 87]
Director of care/nursing	9		[25, 29, 35, 36, 66, 82, 83, 86, 87]
Dietary	8	[45]	[29, 35, 54, 58, 66, 69, 77]
Owner	4		[35, 43, 70, 82]
Pharmacist	4	[53]	[41, 84, 86]

For clarity, studies have been separated into those that mention one or two occupational groups, and studies that mention three or more occupational groups

Evaluation of change at staff or organisational level included the assessment of work life, well-being or satisfaction [65], staff learning or confidence [24, 25, 33, 40, 90], and adaptation or adoption of care processes or protocols [38, 51, 57, 66, 67, 75, 84, 85]. Specifically, the following process measures were assessed: hourly rounding [26], care planning [29], collaborative practice [68]. Finally, one study described changes to the care home (social) environment, such as mealtime ambience [23]. Overall, these data indicate that quality improvement strategies can be successfully implemented in care home settings, but do not differentiate between various quality improvement strategies applied.

The resident-facing interventions delivered as part of QI focused on management of the following: falls (*n* = 16), pressure ulcers (*n* = 9), pain (*n* = 8), medication management and polypharmacy (*n* = 5), nutrition (*n* = 2), incontinence (*n* = 6), end-of-life care (*n* = 5), dyspnoea and pneumonia (*n* = 2), depression (*n* = 1) and heart failure (*n* = 1). Five papers focused on comprehensive multimodal assessment which was similar in nature to Comprehensive Geriatric Assessment (CGA) [96], although it was not always explicitly labelled as such. Twenty-one studies used data from Minimum Dataset (MDS) as an outcome measure. MDS is a system of assessing resident needs and is used for quality assurance and payment of care homes. It was developed in the USA, where it is now mandated, and it is used in Canada and many European countries. The majority of these studies were from the USA [19], with two from Canada. It was often difficult to elucidate the precise details of many resident-facing interventions deployed as part of QI, with no use of standardised reporting frameworks (e.g. TIDIER [97] or EPOC [98]).

Analysing the above factors indicates that there is no pattern or association between the type of QI strategy and the staff groups involved, or the resident-facing intervention. To illustrate this, the following analysis describes one QI strategy, audit and feedback. 9 of the 14 studies involved nursing staff, and 6 involved care assistants with several other occupational groups involved in many studies. Studies described resident-facing interventions which addressed clinical topics such as falls [37, 44, 77], end-of-life care [76, 94], incontinence [81], depression [28], and medication [33]. Staff-level changes reported for audit and feedback included the following: increased self-rated staff competency [28], improved staff interactions and relationships with residents [27], improvement in quality indicators [49]. Finally, for studies of audit and feedback, resident outcomes reported include decrease in hospitalisation [93], decrease in antipsychotic drug prescribing [33], and improvement in end-of-life care quality measures [94]. In summary, there was no evidence that a particular QI strategy had been chosen to address a particular resident problem. Furthermore, there was no pattern of a particular QI strategy being applied to a particular occupational group.

## Discussion

The main finding of this review is that there is a sizeable and increasing body of literature, mostly based in the USA, describing quality improvement (QI) initiatives in care homes settings. The literature predominantly focused on QI interventions at an organisational level, with a smaller literature reporting resident-level process or health outcome metrics, and an even smaller number of articles reporting both organisational-level and resident-level outcomes. Much of the work was descriptive, but the value of descriptions was limited by the lack of reporting according to standardised checklists for QI or resident-level interventions. In many articles, whilst components of change management were specified, such as education or care pathways, the quality improvement strategy was not explicitly stated. There was no association of the type of QI approach with the clinical issue being addressed, neither was there a pattern of the type of QI approach applied to certain occupational groups.

The strengths of this review relate to the structured approach to the literature using both academic and grey literature databases, the inclusive search terms used, and the way in which we separated out quality improvement strategies (change management) from resident-level outcomes in our analysis. A consequence of the lack of statements of quality improvement strategy is that much of the literature uncovered here will have been missed in previous systematic reviews with a focus on a particular quality improvement strategy, for example those focussing just on quality improvement collaboratives [20]. The weaknesses of our approach relate to the fact that much QI work appears in grey literature that may have been beyond the reach of the databases we consulted. Another weakness is the fact that the breadth of the literature retrieved precluded structured approaches to quality appraisal or risk of bias. Such quality appraisal is not usually, though, part of scoping reviews [99], and the variability with which interventions were reported would have challenged systematic review approaches.

Reporting QI initiatives is not easy. To do so comprehensively, authors must report on the change management, and also describe resident interventions and outcomes. To do so within the editorial limitations of a journal article is challenging and this may be reflected in the six QI interventions included here where the authors chose to describe intervention development and evaluation over multiple papers [12, 23, 27, 44, 77, 92]. The SQUIRE checklist [100] is relatively recent (2016) and was published after many of the papers included in our review and this may explain why many authors did not adhere to this reporting guideline. TiDIER [97] and EPOC [98] come from the academic disciplines of clinical trials and systematic reviews, respectively, and may not be well known to the clinical and QI communities. We suggest that, from our experience reviewing these articles, the use of such structured reporting would add considerable clarity.

An important care home-specific consideration which we identified in the literature was that most facilitation of QI came from outside the care home sector, with relatively little evidence of efforts to generate QI expertise within care home staff. There are, though, a number of care home-specific contextual factors which can influence the impact of improvement interventions [101] and a much larger literature suggesting that interventions work in care homes only when they enlist the full support of care home staff [102]. We propose that this is required to develop QI expertise and capacity amongst care home staff.

This work is important to the readership of European Geriatric Medicine because some—such as elderly care physicians in the Netherlands [103]—may already be directly involved in supporting improvement work in care homes. In other instances, such as in the UK, geriatricians and allied health professionals have been recruited to provide leadership around improvement in care homes. It is important for these professionals to understand the uncertainties in the evidence base for the work they are being asked to do.

In conclusion, the literature demonstrates a growing interest in QI in care homes across a number of countries. However, there is a tendency for QI to be reported in vague terms, making the work difficult to understand or synthesise. This in turn makes it difficult for those within the sector to replicate work described in reports. We advocate for a more robust approach to reporting QI interventions in care homes, with attention to describing both the quality improvement strategy (change management), how it leads to improved processes of resident-level care and finally to health outcomes. More attention is required to describe outcomes of QI projects, particularly how they change outcomes for residents. There is limited evidence of efforts to upskill care home staff in QI and this should be a specific focus of future initiatives.

## Electronic supplementary material

Below is the link to the electronic supplementary material.

Supplementary file1 Appendix 1. Search strategy for CINAHL. Appendix 2. Data extraction form. Appendix 3. Reporting Checklist: PRISMA—Extended Scoping (DOCX 29 kb)

## Data Availability

Literature review which relates to publicly accessible documents.

## References

[1] Laing and Buisson (2016). Care of older people—UK market report.

[2] Skills for Care (2019). Care homes with nursing in the adult social care sector.

[3] Skills for Care (2019). Care only homes in the adult social care sector.

[4] Sanford A, Orrell M, Tolson D, Abbatecola A, Arai H, Bauer J (2015). An international definition for “nursing home”. J Am Med Dir Assoc.

[5] Gordon AL, Franklin M, Bradshaw L, Logan P, Elliott R, Gladman JRF (2014). Health status of UK care home residents: a cohort study. Age Ageing.

[6] Bebbington A, Darton R, Bartholomew R, Netten A (2000) Survey of admissions to residential and nursing home care: final report of the 42 month follow-up. PSSRU, Kent

[7] Iliffe S, Davies SL, Gordon AL, Schneider J, Dening T, Bowman C (2016). Provision of NHS generalist and specialist services to care homes in England: review of surveys. Prim Health Care Res Dev.

[8] Robbins I, Gordon A, Dyas J, Logan P, Gladman J (2013). Explaining the barriers to and tensions in delivering effective healthcare in UK care homes: a qualitative study. BMJ Open.

[9] O’Neill D, Briggs R, Holmerova I, Samuelsson O, Gordon AL, Martin FC (2020). COVID-19 highlights the need for universal adoption of standards of medical care for physicians in nursing homes in Europe. Eur Geriatr Med..

[10] Oliver D, Foot C, Humphries R (2014). Making our health and care systems fit for an ageing population.

[11] England NHS (2019). Long term plan.

[12] Marshall M, Pfeifer N, de Silva D, Wei L, Anderson J, Cruickshank L (2018). An evaluation of a safety improvement intervention in care homes in England: a participatory qualitative study. J R Soc Med.

[13] Devi R, Meyer J, Banerjee J, Goodman C, Gladman JRF, Dening T (2018). A quality improvement collaborative aiming for Proactive HEAlthcare of Older People in Care Homes (PEACH): a realist evaluation protocol. BMJ Open..

[14] Giebel C, Harvey D, Akpan A, Chamberlain P (2020). Reducing hospital admissions in older care home residents: a 4-year evaluation of the care home innovation Programme (CHIP). BMC Health Serv Res.

[15] Marshall M, de Silva D, Cruickshank L, Shand J, Wei L, Anderson J (2016). What we know about designing an effective improvement intervention (but too often fail to put into practice). BMJ Qual Saf.

[16] Achterberg WP, Everink IH, van der Steen JT, Gordon AL (2019). We’re all different and we’re the same: the story of the European nursing home resident.

[17] McPheeters M, Kripalani S, Peterson NB, Idowu RT, Jerome RN, Potter SA (2012). Closing the quality gap: revisiting the state of the science (vol. 3: quality improvement interventions to address health disparities). Agency Healthc Res Qual.

[18] Burton JK, Quinn TJ, Gordon AL, MacLullich AMJ, Reynish EL, Shenkin SD (2017). Identifying published studies of care home research: an international survey of researchers. J Nurs Home Res Sci.

[19] Gordon AL, Logan PA, Jones RG, Forrester-Paton C, Mamo JP, Gladman JRF (2012). A systematic mapping review of Randomized Controlled Trials (RCTs) in care homes. BMC Geriatr.

[20] Wells S, Tamir O, Gray J, Naidoo D, Bekhit M, Goldmann D (2018). Are quality improvement collaboratives effective? A systematic review. BMJ Qual Saf.

[21] Taylor MJ, McNicholas C, Nicolay C, Darzi A, Bell D, Reed JE (2014). Systematic review of the application of the plan–do–study–act method to improve quality in healthcare. BMJ Qual Saf.

[22] Tricco AC, Lillie E, Zarin W, O'Brien KK, Colquhoun H, Levac D (2018). PRISMA extension for scoping reviews (PRISMA-ScR): checklist and explanation. Ann Intern Med.

[23] Cranley LA, Hoben M, Yeung J, Estabrooks CA, Norton PG, Wagg A (2018). SCOPEOUT: sustainability and spread of quality improvement activities in long-term care- a mixed methods approach. BMC Health Serv Res.

[24] Little S, Rodgers G, Fitzpatrick JM (2019). Managing deterioration in older adults in care homes: a quality improvement project to introduce an early warning tool. Br J Commun Nurs.

[25] Kezirian AC, McGregor MJ, Stead U, Sakaluk T, Spring B, Turgeon S (2018). Advance care planning in the nursing home setting: a practice improvement evaluation. J Soc Work End Life Palliat Care.

[26] Mitchell RA (2017). Hourly rounding: a fall prevention strategy in long-term care [Developmental Psychology 2800].

[27] Hartmann CW, Mills WL, Pimentel CB, Palmer JA, Allen RS, Zhao S (2018). Impact of intervention to improve nursing home resident-staff interactions and engagement. Gerontologist.

[28] Chodosh J, Price RM, Cadogan MP, Damron-Rodriguez J, Osterweil D, Czerwinski A (2015). A practice improvement education program using a mentored approach to improve nursing facility depression care-preliminary data. J Am Geriatr Soc.

[29] Olsho LEW, Spector WD, Williams CS, Rhodes W, Fink RV, Limcangco R (2014). Evaluation of AHRQ's on-time pressure ulcer prevention program: a facilitator-assisted clinical decision support intervention for nursing homes. Med Care.

[30] Arling PA, Abrahamson K, Miech EJ, Inui TS, Arling G (2014). Communication and effectiveness in a US nursing home quality-improvement collaborative. Nurs Health Sci.

[31] Kojima G, Bell CL, Tamura B, Davis J, Inaba M, Lorenzo P (2014). Combining quality improvement and geriatrics training: the nursing home polypharmacy outcomes project. Gerontol Geriatr Educ.

[32] Powers J, Gwirtsman H, Erwin S (2014). Psychiatric illness and resident assaults among veterans in long-term care facilities. J Gerontol Nurs.

[33] Watson-Wolfe K, Galik E, Klinedinst J, Brandt N (2014). Application of the antipsychotic use in dementia assessment audit tool to facilitate appropriate antipsychotic use in long term care residents with dementia. Geriatr Nurs.

[34] Gama ZAS, Medina-Mirapeix F, Saturno PJ (2011). Ensuring evidence-based practices for falls prevention in a nursing home setting. J Am Med Dir Assoc.

[35] Castle NG, Bost FS (2009). Perfecting patient care: integrating principles of process redesign in nursing homes. J Appl Gerontol.

[36] Ouslander JG, Perloe M, Givens JH, Kluge L, Rutland T, Lamb G (2009). Reducing potentially avoidable hospitalizations of nursing home residents: results of a pilot quality improvement project. J Am Med Dir Assoc.

[37] Bonner A, MacCulloch P, Gardner T, Wilson C (2008). Implementation of a student-led demonstration project on fall prevention in a long-term care facility. Geriatr Nurs.

[38] Lynn J, West J, Hausmann S, Gifford D, Nelson R, McGann P (2007). Collaborative clinical quality improvement for pressure ulcers in nursing homes. J Am Geriatr Soc.

[39] Nace DA, Hoffman EL, Resnick NM, Handler SM (2007). Achieving and sustaining high rates of influenza immunization among long-term care staff. J Am Med Dir Assoc.

[40] Hanson LC, Reynolds KS, Henderson M, Pickard CG (2005). A quality improvement intervention to increase palliative care in nursing homes. J Palliat Med.

[41] Horner JK, Hanson LC, Wood D, Silver AG, Reynolds KS (2005). Using quality improvement to address pain management practices in nursing homes. J Pain Symptom Manage.

[42] Hofmann MT, Bankes PF, Javed A, Selhat M (2003). Decreasing the incidence of falls in the nursing home in a cost-conscious environment: a pilot study. J Am Med Dir Assoc.

[43] Bloodworth LC, Parenti K, Fralix J, Smith M (2018). Using quality measures for performance improvement in the skilled nursing facility/long-term care setting. Top Geriatr Rehabil.

[44] Francis-Coad J, Etherton-Beer C, Bulsara C, Blackburn N, Chivers P, Hill A-M (2018). Evaluating the impact of a falls prevention community of practice in a residential aged care setting: a realist approach. BMC Health Serv Res.

[45] Wright FAC, Law G, Chu SKY, Cullen JS, Le Couteur DG (2017). Residential age care and domiciliary oral health services: reach-OHT-The development of a metropolitan oral health programme in Sydney. Aust Gerodontol.

[46] Rantz MJ, Popejoy L, Vogelsmeier A, Galambos C, Alexander G, Flesner M (2017). Successfully reducing hospitalizations of nursing home residents: results of the missouri quality initiative. J Am Med Dir Assoc.

[47] Rask KJ, Hodge J, Kluge L (2017). Impact of contextual factors on interventions to reduce acute care transfers II implementation and hospital readmission rates. J Am Med Dir Assoc.

[48] Fitzler S, Raia P, Buckley FO, Wang M (2016). Does nursing facility use of habilitation therapy improve performance on quality measures?. Am J Alzheimer's Disease Other Dement.

[49] Eliopoulos C (2013). Affecting culture change and performance improvement in medicaid nursing homes: the promote understanding, leadership, and learning (PULL) program. Geriatr Nurs.

[50] MacLaurin A, McConnell H (2011). Utilizing quality improvement methods to prevent falls and injury from falls: enhancing resident safety in long-term care. J Saf Res.

[51] Glouberman S, Richards J, El Bestawi M, Seidman-Carlson R, Teperman L (2007). Reconnecting to care: a nursing initiative at the baycrest geriatric health system. Nurs Leadersh (Tor Ont).

[52] Barton C, Miller B, Yaffe K (2006). Improved evaluation and management of cognitive impairment: results of a comprehensive intervention in long-term care. J Am Med Dir Assoc.

[53] Munir J, Wright RJ, Carr DB (2006). A quality improvement study on calcium and vitamin D supplementation in long-term care. J Am Med Dir Assoc.

[54] Willingham N (2005). Developing an end-of-life program for long term care residents. Jt Comm J Qual Patient Saf.

[55] Abel RL, Warren K, Bean G, Gabbard B, Lyder CH, Bing M (2005). Quality improvement in nursing homes in Texas: results from a pressure ulcer prevention project. J Am Med Dir Assoc.

[56] Wagner LM, Capezuti E, Taylor JA, Sattin RW, Ouslander JG (2005). Impact of a falls menu-driven incident-reporting system on documentation and quality improvement in nursing homes. Gerontologist.

[57] Baier RR, Gifford DR, Lyder CH, Schall MW, Funston-Dillon DL, Lewis JM (2003). Quality improvement for pressure ulcer care in the nursing home setting: the northeast pressure ulcer project. J Am Med Dir Assoc.

[58] Boyle PJ, O'Neil KW, Berry CA, Stowell SA, Miller SC (2013). Improving diabetes care and patient outcomes in skilled-care communities: successes and lessons from a quality improvement initiative. J Am Med Dir Assoc.

[59] Ouslander JG, Bonner A, Herndon L, Shutes J (2014). The interventions to reduce acute care transfers (INTERACT) quality improvement program: an overview for medical directors and primary care clinicians in long term care. J Am Med Dir Assoc.

[60] Törmä J, Winblad U, Saletti A, Cederholm T (2018). The effects of nutritional guideline implementation on nursing home staff performance: a controlled trial. Scand J Caring Sci.

[61] Francis-Coad J, Etherton-Beer C, Bulsara C, Nobre D, Hill A-M (2016). Using a community of practice to evaluate falls prevention activity in a residential aged care organisation: a clinical audit. Aust Health Rev Publ Aust Hosp Assoc.

[62] Unroe KT, Nazir A, Holtz LR, Maurer H, Miller E, Hickman SE (2015). The optimizing patient transfers, impacting medical quality, and improving symptoms: transforming institutional care approach: preliminary data from the implementation of a centers for medicare and medicaid services nursing facility demonstration project. J Am Geriatr Soc.

[63] Vikstrom S, Sandman P-O, Stenwall E, Bostrom A-M, Saarnio L, Kindblom K (2015). A model for implementing guidelines for person-centered care in a nursing home setting. Int Psychogeriatr.

[64] Boelsma F, Baur VE, Woelders S, Abma TA (2014). "Small" things matter: residents involvement in practice improvements in long-term care facilities. J Aging Stud.

[65] Norton P, Cranley L, Cummings G, Estabrooks C (2013). Report of a pilot study of quality improvement in nursing homes led by healthcare aides. Eur J Person Centered Healthc.

[66] Sharkey S, Hudak S, Horn SD, Barrett R, Spector W, Limcangco R (2013). Exploratory study of nursing home factors associated with successful implementation of clinical decision support tools for pressure ulcer prevention. Adv Skin Wound Care.

[67] Dolansky MA, Hitch JA, Pina IL, Boxer RS (2013). Improving heart failure disease management in skilled nursing facilities: lessons learned. Clin Nurs Res.

[68] Puxty J, Brander RA, Murphy S, Byrnes V (2012). Promoting quality improvement in long-term care: a multi-site collaboration to improve outcomes with pneumonia, falls, bacteriuria and behavioural issues in dementia. Healthc Q.

[69] Long C, Morgan BM, Alonzo TR, Mitchell KM, Bonnell DK, Beardsley ME (2010). Improving pain management in long-term care: the campaign against pain. J Hosp Palliat Nurs.

[70] Rantz MJ, Flesner MK, Zwygart-Stauffacher M (2010). Improving care in nursing homes using quality measures/indicators and complexity science. J Nurs Care Qual.

[71] Baier RR, Gifford DR, Patry G, Banks SM, Rochon T, DeSilva D (2004). Ameliorating pain in nursing homes: a collaborative quality-improvement project. J Am Geriatr Soc.

[72] Marshall M, Cruickshank L, Shand J, Perry S, Anderson J, Wei L (2017). Assessing the safety culture of care homes: a multimethod evaluation of the adaptation, face validity and feasibility of the manchester patient safety framework. BMJ Qual Saf.

[73] Dupler AM, Crogan NL, Short R (2001). Pathways to quality improvement for boarding homes: a washington state model. J Nurs Care Qual.

[74] Bakerjian D, Bonner A, Benner C, Caswell C, Weintraub A, Koren MJ (2011). Reducing perceived barriers to nursing homes data entry in the advancing excellence campaign: the role of LANEs (local area networks for excellence). J Am Med Dir Assoc.

[75] Leone AF, Standoli F, Hirth V (2009). Implementing a pain management program in a long-term care facility using a quality improvement approach. J Am Med Dir Assoc.

[76] Boogaard JA, de Vet HCW, van Soest-Poortvliet MC, Anema JR, Achterberg WP, van der Steen JT (2018). Effects of two feedback interventions on end-of-life outcomes in nursing home residents with dementia: a cluster-randomized controlled three-armed trial. Palliat Med.

[77] Colon-Emeric CS, McConnell E, Pinheiro SO, Corazzini K, Porter K, Earp KM (2013). CONNECT for better fall prevention in nursing homes: results from a pilot intervention study. J Am Geriatr Soc.

[78] Sheaff R, Sherriff I, Hennessy CH (2018). Evaluating a dementia learning community: exploratory study and research implications. BMC Health Serv Res.

[79] Kane RL, Huckfeldt P, Tappen R, Engstrom G, Rojido C, Newman D (2017). Effects of an intervention to reduce hospitalizations from nursing homes: a randomized implementation trial of the INTERACT program. JAMA Int Med.

[80] Boyd M, Armstrong D, Parker J, Pilcher C, Zhou L, McKenzie-Green B (2014). Do gerontology nurse specialists make a difference in hospitalization of long-term care residents? results of a randomized comparison trial. J Am Geriatr Soc.

[81] Goodman C, Davies LS, Norton C, Fader M, Morris J, Wells M (2013). Can district nurses and care home staff improve bowel care for older people using a clinical benchmarking tool?. Br J Comm Nurs.

[82] Rantz MJ, Zwygart-Stauffacher M, Hicks L, Mehr D, Flesner M, Petroski GF (2012). Randomized multilevel intervention to improve outcomes of residents in nursing homes in need of improvement. J Am Med Dir Assoc.

[83] Rantz MJ, Popejoy L, Petroski GF, Madsen RW, Mehr DR, Zwygart-Stauffacher M (2001). Randomized clinical trial of a quality improvement intervention in nursing homes. Gerontologist.

[84] Colón-Emeric C, Schenck A, Gorospe J, McArdle J, Dobson L, DePorter C (2006). Translating evidence-based falls prevention into clinical practice in nursing facilities: results and lessons from a quality improvement collaborative. J Am Geriatr Soc.

[85] Rask K, Parmelee PA, Taylor JA, Green D, Brown H, Hawley J (2007). Implementation and evaluation of a nursing home fall management program. J Am Geriatr Soc.

[86] Kaasalainen S, Brazil K, Akhtar-Danesh N, Coker E, Ploeg J, Donald F (2012). The evaluation of an interdisciplinary pain protocol in long term care. J Am Med Dir Assoc.

[87] Rolland Y, Mathieu C, Piau C, Cayla F, Bouget C, Vellas B (2016). Improving the quality of care of long-stay nursing home residents in France. J Am Geriatr Soc.

[88] Winters S, Kool RB, Klazinga NS, Huijsman R (2014). The influence of corporate structure and quality improvement activities on outcome improvement in residential care homes. Int J Qual Health Care.

[89] Masso M, Westera A, Quinsey K, Morris D, Pearse J (2011). Encouraging best practice in residential aged care program: final evaluation report. Centre for health service development.

[90] Cranley LA, Norton PG, Cummings GG, Barnard D, Estabrooks CA (2011). SCOPE: Safer care for older persons (in residential) environments: a study protocol. Implement Sci IS.

[91] Anderson RA, Corazzini K, Porter K, Daily K, McDaniel RR, Colon-Emeric C (2012). CONNECT for quality: protocol of a cluster randomized controlled trial to improve fall prevention in nursing homes. Implement Sci IS.

[92] Huckfeldt PJ, Kane RL, Yang Z, Engstrom G, Tappen R, Rojido C (2018). Degree of implementation of the interventions to reduce acute care transfers (INTERACT) quality improvement program associated with number of hospitalizations. J Am Geriatr Soc.

[93] Ouslander JG, Lamb G, Tappen R, Herndon L, Diaz S, Roos BA (2011). Interventions to reduce hospitalizations from nursing homes: evaluation of the INTERACT II collaborative quality improvement project. J Am Geriatr Soc.

[94] Keay TJ, Alexander C, McNally K, Crusse E, Eger RE (2003). Nursing home physician educational intervention improves end-of-life outcomes. J Palliat Med.

[95] Hartmann CW, Palmer JA, Mills WL, Pimentel CB, Allen RS, Wewiorski NJ (2017). Adaptation of a nursing home culture change research instrument for frontline staff quality improvement use. Psychol Serv.

[96] Chadborn NH, Goodman C, Zubair M, Sousa L, Gladman JRF, Dening T (2019). Role of comprehensive geriatric assessment in healthcare of older people in UK care homes: realist review. BMJ Open.

[97] Hoffmann TC, Glasziou PP, Boutron I, Milne R, Perera R, Moher D (2014). Better reporting of interventions: template for intervention description and replication (TIDieR) checklist and guide. BMJ.

[98] Mowatt G, Grimshaw JM, Davis DA, Mazmanian PE (2001). Getting evidence into practice: the work of the Cochrane Effective Practice and Organization of Care Group (EPOC). J Contin Educ Health Prof.

[99] Grant MJ, Booth A (2009). A typology of reviews: an analysis of 14 review types and associated methodologies. Health Inf Libr J.

[100] Ogrinc G, Davies L, Goodman D, Batalden P, Davidoff F, Stevens D (2015). SQUIRE 2.0 (Standards for QUality Improvement Reporting Excellence): revised publication guidelines from a detailed consensus process. Am J Crit Care.

[101] Bunn F, Goodman C, Corazzini K, Sharpe R, Handley M, Lynch J (2020). Setting priorities to inform assessment of care homes’ readiness to participate in healthcare innovation: a systematic mapping review and consensus process. Int J Environ Res Publ Health.

[102] Davies SL, Goodman C, Bunn F, Victor C, Dickinson A, Iliffe S (2011). A systematic review of integrated working between care homes and health care services. BMC Health Serv Res.

[103] Poot AJ, de Waard CS, Wind AW, Caljouw MAA, Gussekloo J (2017). A structured process description of a pragmatic implementation project: improving integrated care for older persons in residential care homes. Inquiry.

