# Supplementary material for: Quality improvement in long-term care settings: a scoping review of effective strategies used in care homes
Source: Eur Geriatr Med. 2020 Sep 4;12(1):17–26. doi: 10.1007/s41999-020-00389-w (PMC7472942; doi:10.1007/s41999-020-00389-w)
Supplement: Supplementary file 1 — Supplementary file1 Appendix 1. Search strategy for CINAHL. Appendix 2. Data extraction form. Appendix 3. Reporting Checklist: PRISMA—Extended Scoping (DOCX 29 kb) [file 41999_2020_389_MOESM1_ESM.docx]

# Supplementary material

Quality improvement in long term care settings: a scoping review of effective strategies used in care homes

Neil H Chadborn (1,2) 0000-0003-1368-7983

Reena Devi (3) 0000-0003-2834-8597

Kathryn Hinsliff-Smith (4) 0000-0003-4259-3118

Jay Banerjee (5) 0000-0001-9275-4755

Adam Gordon (1,2) 0000-0003-1676-9853

1. Division of Medical Science and Graduate Entry Medicine, School of Medicine, University of Nottingham, UK
2. NIHR Applied Research Collaboration East Midlands, UK
3. School of Healthcare, University of Leeds, Leeds, UK
4. Faculty of Health and Life Sciences, De Montfort University, Leicester, UK
5. University Hospitals of Leicester NHS Trust, UK

Corresponding author email address: [Neil.Chadborn@nottingham.ac.uk](mailto:Neil.Chadborn@nottingham.ac.uk)

# Appendix 1 Search strategy for CINAHL

Interface - EBSCOhost Research Databases
Search Screen - Advanced Search
Database - CINAHL Plus with Full Text

| S1 | MW homes for the aged OR MW long term care OR MW residential home OR MW residential facility OR MW Institutional care OR MW snf OR MW ltcf OR MW skilled nursing facility OR MW nursing home OR MW care home | Search modes - Boolean/Phrase | 69,647 |
| --- | --- | --- | --- |
| S2 | MW quality improvement OR MW total quality management OR MW pdsa OR MW process improvement | Search modes - Boolean/Phrase | 44,454 |
| S3 | (MW quality improvement OR MW total quality management OR MW pdsa OR MW process improvement) AND (S1 AND S2) | Search modes - Boolean/Phrase | 1,932 |
| S4 | (MW quality improvement OR MW total quality management OR MW pdsa OR MW process improvement) AND (S1 AND S2) | Narrow by Language: - english  Search modes - Boolean/Phrase | 1,911 |
| S5 | (MW quality improvement OR MW total quality management OR MW pdsa OR MW process improvement) AND (S1 AND S2) | Narrow by SubjectAge: - all adult  Narrow by Language: - english  Search modes - Boolean/Phrase | 576 |

# Appendix 2 Data extraction form

| Author |  | Response options | |
| --- | --- | --- | --- |
| Year of publication | | |  |
| 1 | Details of study type | |  |
|  |  | Experimental, quantitative with control | Quantitative Quality Improvement |
|  |  | Qualitative Quality Improvement study | Observational |
| 2 | Protocol or preparatory | |  |
| Details about quality improvement initiative | | |  |
| 3 | Number of participants of QI initiative | |  |
| 4 | Professional groups | |  |
|  |  | Nursing/advanced nurses | Pharmacist |
|  |  | Care home manager/owner | Social worker |
|  |  | Physio or therapists incl nutrition | Dietary |
|  |  | Doctors | Interprofessional |
|  |  | Admin | Resident |
|  |  | Care assistants | Not reported |
| 5 | Country of authors | |  |
|  |  | US | Spain |
|  |  | UK | Australia |
|  |  | Netherlands | New Zealand |
|  |  | Canada | Sweden |
|  |  | Norway | France |
| 6 | Type of QI initiative | |  |
|  |  | Clinical education | Involving resident |
|  |  | Training on QI methods | Communication |
|  |  | Pathways | QIC |
|  |  | Data analysis | Peer or champions |
|  |  | Audit or evaluation & feedback | Diffussion of innovation |
|  |  | Teams, MDT | Specific improvement 'brand' |
|  |  | PDSA or iterative | Not reported or NA |
| 7 | Facilitator of QI | |  |
|  |  | External academic | External not specified |
|  |  | External QI consultant | Internal |
| 8 | Comparator | |  |
| 9 | Outcomes of QI | |  |
| Resident level intervention | | |  |
| 10 | Number of participants - residents | |  |
| 11 | Resident-level intervention (&comparator if not usual care) | |  |
| 12 | Condition or syndrome addressed | |  |
|  |  | Falls | Dementia management |
|  |  | Pressure Ulcers | Physical restraint |
|  |  | Incontinence | Respiratory |
|  |  | Depression | Medication & nutrition |
|  |  | Pain | Heart failure |
|  |  | End of Life Care | Comprehensive |
| 13 | Outcome measures | |  |
| 14 | Reported findings of study | |  |
| 15 | Type of outcome reported | |  |
|  |  | Process measure  (e.g. pain relief given) | QoL |
|  |  | Incidence or prevalence | Satisfaction of resident or family |
|  |  | Clinical measure or episodes or number of events  (e.g. falls or incontinence) |  |
| 16 | Quality of study or flaws | |  |
| 17 | Vested interests of authors | |  |

# Appendix 3 Reporting Checklist: PRISMA – Extended Scoping

| Section | Item |  |
| --- | --- | --- |
| Title |  | Title includes ‘scoping review’ |
| Abstract | 1. Structured summary | Structured abstract that includes aims, databases searched, key findings and conclusions |
| Introduction | 1. Rationale | Background literature which describes the knowledge gap |
|  | 1. Objectives | Aim of review is stated, and the diversity of QI approaches and recent implementation in care homes justifies the scoping method |
| Methods | 1. Protocol & registration | Protocol was not published or registered |
|  | 1. Eligibility criteria | Definition of QI, search strategy and selection criteria are described |
|  | 1. Information sources | Databased searched are listed |
|  | 1. Search | Full search strategy for CINAHL database is shown in Appendix 1. |
|  | 1. Selection of sources | Screening and selection are described including team input and consensus |
|  | 1. Data charting process | Description of author team extracting data in duplicate into spreadsheet |
|  | 1. Data items | Data extraction form shown in Appendix 2. Data is shown in Table 1 and described in the text |
|  | 1. Critical appraisal | Data extraction included identification of bias or flawed methods, also potential conflict of interests |
|  | 1. NA for scoping |  |
|  | 1. Synthesis | Descriptive synthesis |
|  | 1. NA for scoping |  |
|  | 1. NA for scoping |  |
| Results | 1. Selection of sources | Flow diagram, Fig 1 |
|  | 1. Characteristics | Methods and type of QI is described |
|  | 1. Critical appraisal | Biases or flaws are noted in text |
|  | 1. Results of individual sources |  |
|  | 1. Synthesis | Descriptive summary of articles in text |
|  | 1. NA for scoping |  |
|  | 1. NA for scoping |  |
| Discussion | 1. Summary | Key messages |
|  | 1. Limitations | Limitations of method and difficulty of summarizing diverse literature is noted |
|  | 1. Conclusions | Interpretation of results is given |
| Funding | 27. | Funding source acknowledged |
